# Fibroblast growth factor receptor fusions in cancer: opportunities and challenges

**DOI:** 10.1186/s13046-021-02156-6

**Published:** 2021-11-03

**Authors:** Lingfeng Chen, Yanmei Zhang, Lina Yin, Binhao Cai, Ping Huang, Xiaokun Li, Guang Liang

**Affiliations:** 1 Clinical Pharmacy Center, Department of Pharmacy, Zhejiang Provincial People’s Hospital, Affiliated People’s Hospital, Hangzhou Medical College, Hangzhou, 310014 Zhejiang China; 2 School of Pharmaceutical Sciences, Hangzhou Medical College, Hangzhou, 310012 Zhejiang China; 3 Chemical Biology Research Center, School of Pharmaceutical Sciences, Wenzhou Medical University, Wenzhou, 325035 Zhejiang China

**Keywords:** Fibroblast growth factor receptors, Fusion proteins, Chromosomal translocation, Cancer, Inhibitors

## Abstract

Fibroblast growth factors (FGFs) and their receptors (FGFRs) play critical roles in many biological processes and developmental functions. Chromosomal translocation of FGFRs result in the formation of chimeric FGFR fusion proteins, which often cause aberrant signaling leading to the development and progression of human cancer. Due to the high recurrence rate and carcinogenicity, oncogenic FGFR gene fusions have been identified as promising therapeutic targets. Erdafitinib and pemigatinib, two FGFR selective inhibitors targeting FGFR fusions, have been approved by the U.S. Food and Drug Administration (FDA) to treat patients with urothelial cancer and cholangiocarcinoma, respectively. Futibatinib, a third-generation FGFR inhibitor, is under phase III clinical trials in patients with FGFR gene rearrangements. Herein, we review the current understanding of the FGF/FGFRs system and the oncogenic effect of FGFR fusions, summarize promising inhibitors under clinical development for patients with FGFR fusions, and highlight the challenges in this field.

## Background

Structural chromosome rearrangements between two genes may lead to the deregulation of genes originating from translocation, insertion, inversion, or deletion. Such gene fusions are mostly pathogenic and have offered important insights into carcinogenesis [1]. Enforced dimerization/oligomerization and inactivation of autoinhibition mechanisms are the two key mechanisms that lead to aberrant kinase activity [2]. Growing numbers of fusion proteins encoded by the hybrid genes have been novel targets for personalized cancer therapy that significantly improved patient survival [3, 4]. Imatinib was the first U.S. Food and Drug Administration (FDA) approved drug that was designed to inhibit the oncogenic fusion protein BCR–ABL [5]. The success of imatinib in chronic myeloid leukemia (CML) patients has triggered great interest in developing novel drugs targeting the chimeric proteins, including ALK [6], ROS1 [7], RET [8], MET [9] and NTRK [10] fusions. To date, more than 10 kinase inhibitors have been approved by the FDA for the treatment of fusion-positive cancers.

Fibroblast growth factor (FGF) receptors (FGFRs) are highly conserved single transmembrane receptor tyrosine kinases (RTKs), consisting of an extracellular ligand-binding domain and a cytoplasmic conserved tyrosine kinase domain. FGFR signals play important roles in cellular proliferation and survival, embryonic development, fetal organogenesis, metabolism homeostasis, and tissue repair [11, 12]. Dysregulation of FGFR signaling contributes to oncogenesis and tumour progression, drug resistance to anticancer therapy, as well as the occurrence of immune evasion and angiogenesis in the tumour microenvironment (TME). Aberrant activation of the oncogenic FGFR signaling pathway is mainly caused by the deregulation of FGF ligand and FGFR genetic alterations, including amplification, activating mutations, and gene fusions [12–14].

FGFR fusions occur when the kinase domain of FGFR1–4 fuses with a partner containing a constitutive dimerization/oligomerization motif, thereby activating the signaling in a ligand-independent manner [15–17]. With the advances in deep-sequencing technology, cases of FGFR-related fusion genes are growing exponentially. The dominant oncogenic fusion partners drive malignant initiation and progression, especially in urothelial cancer, cholangiocarcinoma, and glioblastoma [3, 18]. Therefore, certain FGFR fusions have emerged as biomarkers and rational druggable targets.

## FGF–FGFR-HS system

**Fig. 1:**
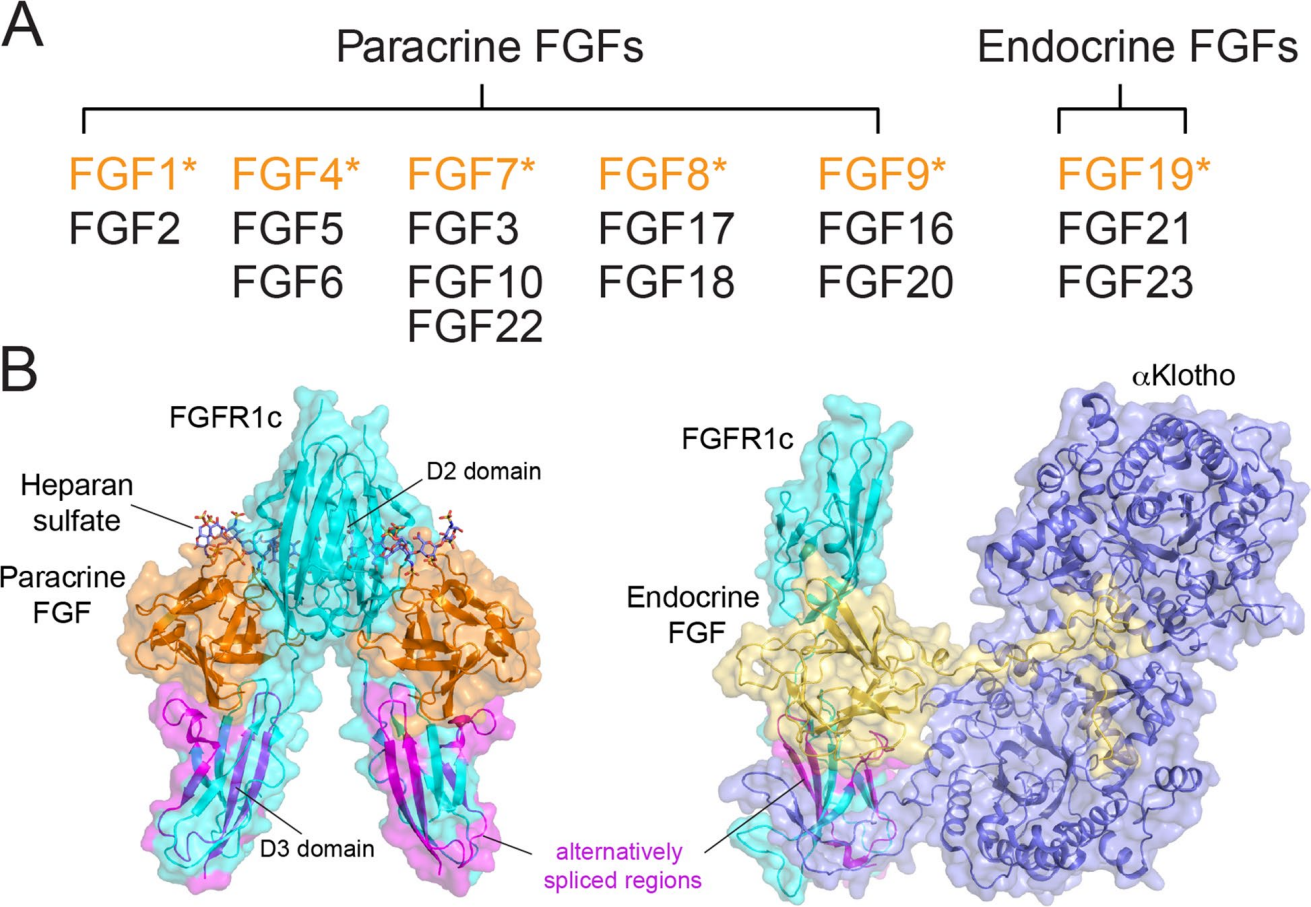
FGF–FGFR-HS system. **A** 18 functional mammalian FGFs sorted into six subfamilies. Each founding members are colored in orange. **B** Left: paracrine FGFs bind to the D2-D3 domains of FGFRs and HS to form 2:2:2 FGF-FGFR-HS complex (PDB: 1FQ9). Right: Endocrine FGF-FGFR-Klotho complex PDB ID: 5 W21). Alternatively-spliced D3 domain of FGFR is highlighted in purple

The human FGF family consists of 18 functional glycoproteins (FGF1–10 and FGF16–23). They are grouped into six subfamilies according to their sequence similarity and phylogeny [11, 19]. FGFs belonging to the FGF1/4/7/8/9 subfamilies act as paracrine ligands, whereas FGF19/21/23 belonging to FGF19 subfamily are endocrine hormones (Fig. 1A) [20]. FGFs exert their function through binding to and dimerizing their cognate receptors. The extracellular ligand-binding segment of FGFR1–4 comprises three immunoglobulin-like domains (D1–D3) [21]. The C-terminal half of D3 domains of FGFR1–3 are encoded by two alternative exons (termed exon IIIb and IIIc), yielding “b” and “c” isoforms. These cell and tissue-specific alternative splicing events converse the sequence of crucial residues in the pocket of the ligands-binding D3 domain of FGFR1–3, thus governing FGF binding specificity and varying signaling patterns [22]. The “c” isoforms of FGFRs are primarily expressed in mesenchymal cells and favorably recognize epithelial-derived FGF1/4/8/9/19 subfamilies, whereas the “b” isoforms are mostly expressed in epithelial tissues, showing rigorous binding specificity for the mesenchymal-expressed FGF7 subfamily [23].

Besides ligands, receptors dimerization is assisted by a cofactor named heparan sulfate proteoglycans (HSPGs) ubiquitously present on the cell surface and in the extracellular matrix. Sulfate group-rich HSPGs interact with the lysine/arginine-rich surface termed “heparan binding site (HBS)” of both FGFs and receptors to stabilize the FGF–FGFR binding interface, thereby promoting the formation of a 2:2:2 quaternary complex of FGF, FGFR, and HS (Fig. 1B) [24]. HSPGs are obligatory cofactors for both paracrine and endocrine FGFs. The endocrine FGF19 subfamily (FGF19/21/23) has less surface-exposed lysine/arginine residues on the HBS than the paracrine FGFs, leading to intrinsically reduced affinity to HSPG binding. Thereby, the FGF19 subfamily acts as hormones released from the expression site into the body circulation system. For endocrine FGFs, a secondary cofactor is necessary to stabilize FGF–FGFR complex (Fig. 1B), i.e., FGF19/21 requires β-Klotho as a secondary cofactor to promote signaling, whereas FGF23 utilizes α-Klotho to form FGF23–FGFR–αKlotho–heparan sulfate quaternary complex [25, 26].

## FGF signaling pathways in cancer

**Fig. 2:**
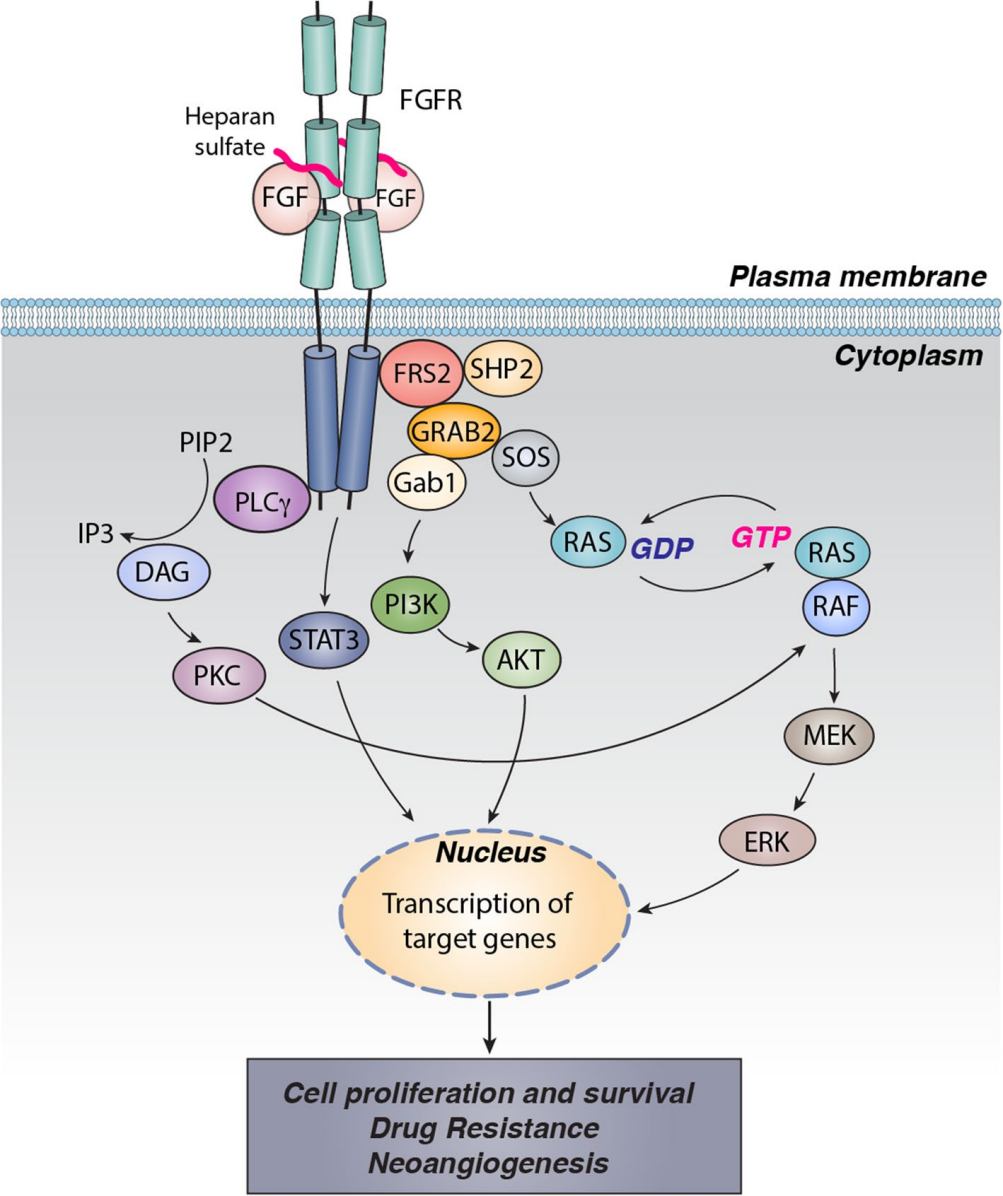
FGFR signaling in cancers. FGF, HSPG, and FGFR form 2:2:2 ternary complex, followed by receptor dimerization and kinase transphosphorylation. FGFR downstream adaptor protein FRS2 interacts with SHP2 and GRB2 complex, leading to subsequent activation of PI3K-AKT and RAS-MEK-ERK signaling pathways. Another FGFR substrate, PLC-g, binds to phosphotyrosine and hydrolyzes PIP2 to generate IP3 and DAG, which in turn activate PKC and MAPK pathway, resulting in cell migration, proliferation, and differentiation. Depending on the cellular context, FGFRs have the capability to activate the JAK-STAT3 signalling pathway. Aberrant FGFR signaling may be induced by (i) increased expression of FGFs (ligand-dependent), or (ii) FGFR alteration, including mutation, amplification or translocation (ligand-independent)

FGF ligands-induced receptor dimerization and tyrosine trans-phosphorylation ultimately generate docking sites for intracellular effector molecules. FGFR substrates 2α (FRS2α) and phospholipase Cγ (PLCγ) are the two substrates that bind directly to the kinase domain [27]. Following phosphorylation, FRS2α and PLCγ will trigger multiple signaling pathways, including RAS–mitogen-activated protein kinase (MAPK), phosphoinositide 3- kinase (PI3K)–protein kinase B (AKT), protein kinase C (PKC), and STAT-dependent signaling, thereby contributing to carcinogenesis by stimulating cancer cell proliferation and survival, neoangiogenesis, and drug resistance (Fig. 2).

FRS2α is a non-enzymatic adaptor protein of FGFR signaling, which functions as the central node for the assembly of various signaling complexes [28, 29]. FRS2α has six tyrosine residues in the long flexible C-terminal tail that can be phosphorylated by activated FGFRs. Specifically, phosphorylation at the four tyrosine residues (Tyr-196, Tyr-306, Tyr-349 and Tyr-392) will enable FRS2α to recruit two preformed growth factor receptor-bound protein 2 (GRB2) containing binary complexes, i.e., GRB2–son of Sevenless (SOS) and GRB2–GRB2-associated-binding protein 1 (GAB1). FRS2α assists the translocation of GRB2–SOS complex to downstream substrate RAS and activates RAS through GTP exchange, followed by activation of MAPK signaling. Upon the recruitment of the GRB2–GAB1 complex, PI3K will be recruited to FRS2α, leading to the translocation and transphosphorylation of AKT [30, 31]. In addition, FRS2α phosphorylation at C-terminal tail (pTyr-436 and pTyr-471) offers a docking site for SH2-containing tyrosine phosphatase (SHP2) and further diversifies FGFR signaling [32, 33].

PLCγ is a hydrolase docking to the phosphorylated tyrosine (Tyr-769 in FGFR2) in the C- terminal tail of FGFR through its cSH2 domain. PLCγ stimulates the release of Ca^2+^ from the endoplasmic reticulum into the cytosol and activates the Ser/Thr kinase protein kinase C (PKC), resulting in cell migration, proliferation and differentiation [34]. The crystal structure of PLCγ cSH2 domain and C-terminal phosphorylated FGFR2 kinase complex has shown the engagement of cSH2 of PLCγ with activated FGFR2 kinase, demonstrating that the recruitment of PLCγ is followed by FGFR dimerization. One activated FGFR kinase functions as the recruiter of PLCγ, whereas the other is responsible for PLCγ phosphorylation at Tyr-771 and Tyr-783 [35].

## Mechanisms of FGFR fusion oncoprotein

### Autoinhibition modes of FGFR

**Fig. 3:**
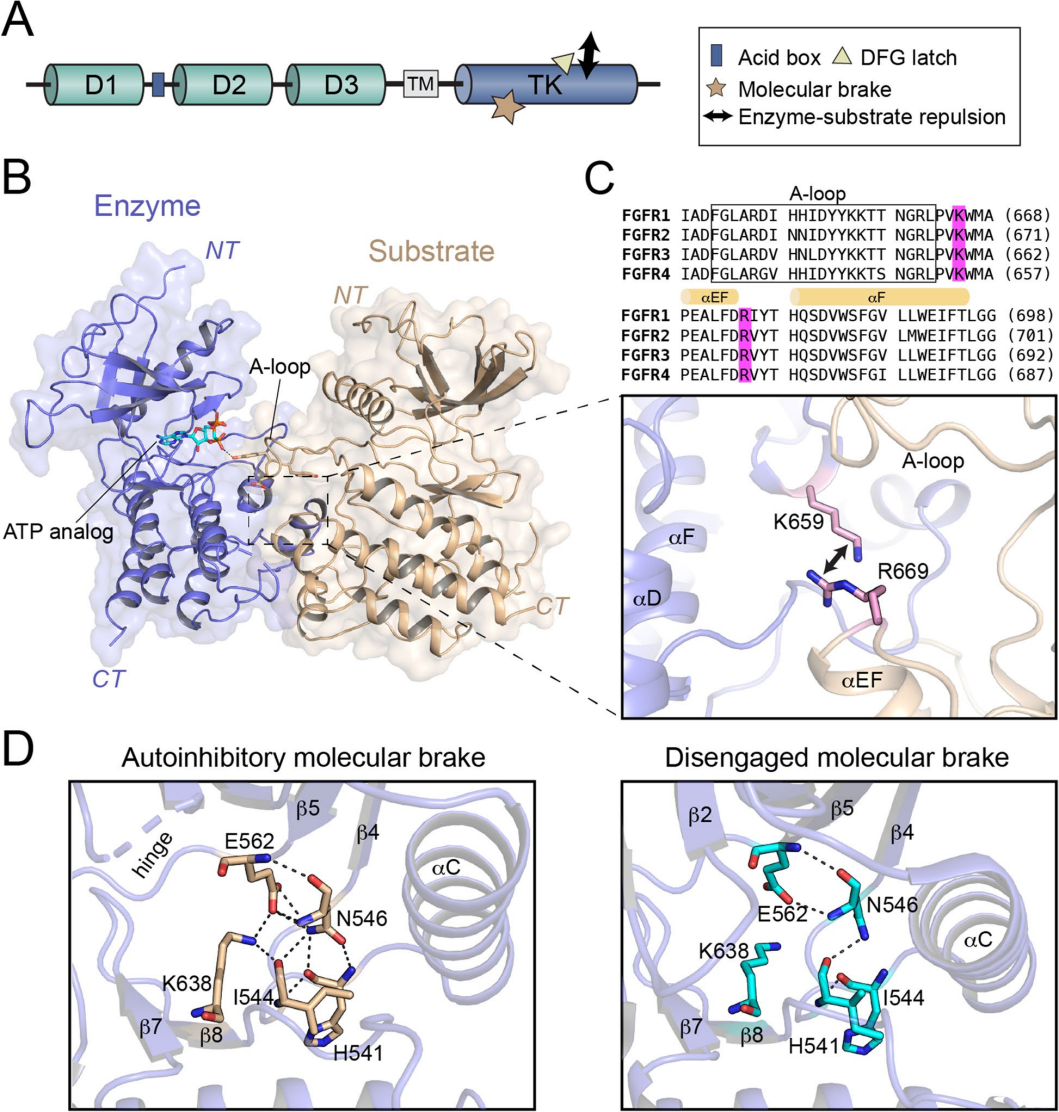
FGFR autoinhibition mechanisms. **A** Schematic representations of FGFR autoinhibition modes consisting of acid box regulation, molecular brake, DFG latch, and the repulsion between enzyme and substrate kinases **B** overall view of the asymmetric FGFR kinase A-loop transphosphorylation complex (PDB: 6PNX). Enzyme- and substrate-acting FGFR kinases are colored in green, blue and wheat, respectively. **C** FGFR3 K659 and R669 form enzyme-substrate electrostatic clash. Sequence alignment of the kinase domains of FGFR1–4 shows the conservation of autoinhibition mechanism. **D** Hydrogen bonding pattern of the autoinhibitory molecular brake in FGFR1 (left, PDB ID: 1fgk) and disengaged brake (right, PDB ID: 3gqi). The dashed lines denote hydrogen bonds

Receptor tyrosine kinase (RTK) signaling is tightly regulated by protein allostery from the extracellular domain or the intracellular tyrosine kinase domains (Fig. 3A) [36]. The first line of FGFR autoinhibition is mediated by the extracellular D1 domain and the acid box subregion between the D1 and D2 domain that prevents inadvertent ligand activation [21]. As in cytoplasmic FGFRs kinase domains, unphosphorylated kinase are more precisely controlled to minimize undesired signaling [37]. Binding of extracellular ligand induces receptor dimerization, thereby forcing the kinase domains into appropriate proximity and orientation for transautophosphorylation on specific tyrosine sites. Accumulated biochemical and structural evidences indicate that autophosphorylation of FGFR kinase occurs in a sequentially and accurately ordered reaction that can be separated into three phases [38]. During the first phase, the tyrosine residues positioned at the activation loop (A-loop) accomplish phosphorylation, which in turn induces the active conformation of the kinase, leading to 50–100-fold upregulation of kinase activity. The second phase includes the phosphorylation of tyrosine residues located in the kinase insert, JM segment, and the carboxy-tail region. In the last phase, FGFR kinase activity is increased by an additional 10-fold via the phosphorylation of the second tyrosine residue within the A-loop (Y654 in FGFR1). Thus, A-loop transphosphorylation is an obligatory step for kinase activation, and therefore is the key mechanism for FGFR kinase autoinhibition [39].

In 2020, the structure of an asymmetric complex of two FGFR3 kinases caught in the act of transphosphorylation was successfully solved [40]. In this complex, one kinase serves as an enzyme, whereas the other is the substrate (Fig. 3B). This is distinct from the activator-receiver relationship of EGFR asymmetric homodimer. The substrate acting kinase offers its first A-loop tyrosine residue (Tyr647 in FGFR3) for the initiation of A-loop tyrosine transphosphorylation reaction. Notably, the FGFR asymmetric kinase dimer is thermodynamically disadvantaged owing to the electrostatic repulsion between their C-lobes, which is mainly caused by the two conserved residues within the kinase domain, i.e., Lys659 in enzyme and Arg669 in substrate kinases, thereby governing the kinase A-loop transphosphorylation event (Fig. 3C) [40]. In addition, an autoinhibitory network of hydrogen bond interactions named ‘molecular brake’ controls the kinase activity to a low-level state. These include Glu562 residue (FGFR1 numbering) in the kinase hinge, Asn546 in the αC-β4 loop, and Lys638 in the β8 strand (Fig. 3D) [41]. Another cluster of hydrophobic interactions centered on the Asp-Phe-Gly (DFG) motif called the ‘DFG latch’ is also involved in FGFR autoinhibition by affecting the conformation of the A-loop as well as the N-lobe rotation [36, 42].

The activation of FGFRs is normally down-regulated by those autoinhibitory mechanisms, and the disruption of autoinhibition promotes various cancers. It is important to find the underlying mechanism of gene fusion-induced autoinhibition release. Firstly, the oncogenic potential of fusions has been attributed to the loss of the critical D1 domain and acid box region of FGFRs [43]. Besides, the energy gain from fusion-induced receptor dimerization may overcome the repulsion between two kinases, thereby promoting the A-loop transphosphorylation and leading to kinase activation [38, 40]. Indeed, analysis of phosphopeptides from human astrocytes expressing FGFR3-TACC3 fusions shows the tyrosine Tyr647 (first A-loop tyrosine) in FGFR3 exhibited the highest enrichment in phosphorylation [17].

### Oncogenic mechanisms of FGFR chimeric proteins

**Fig. 4:**
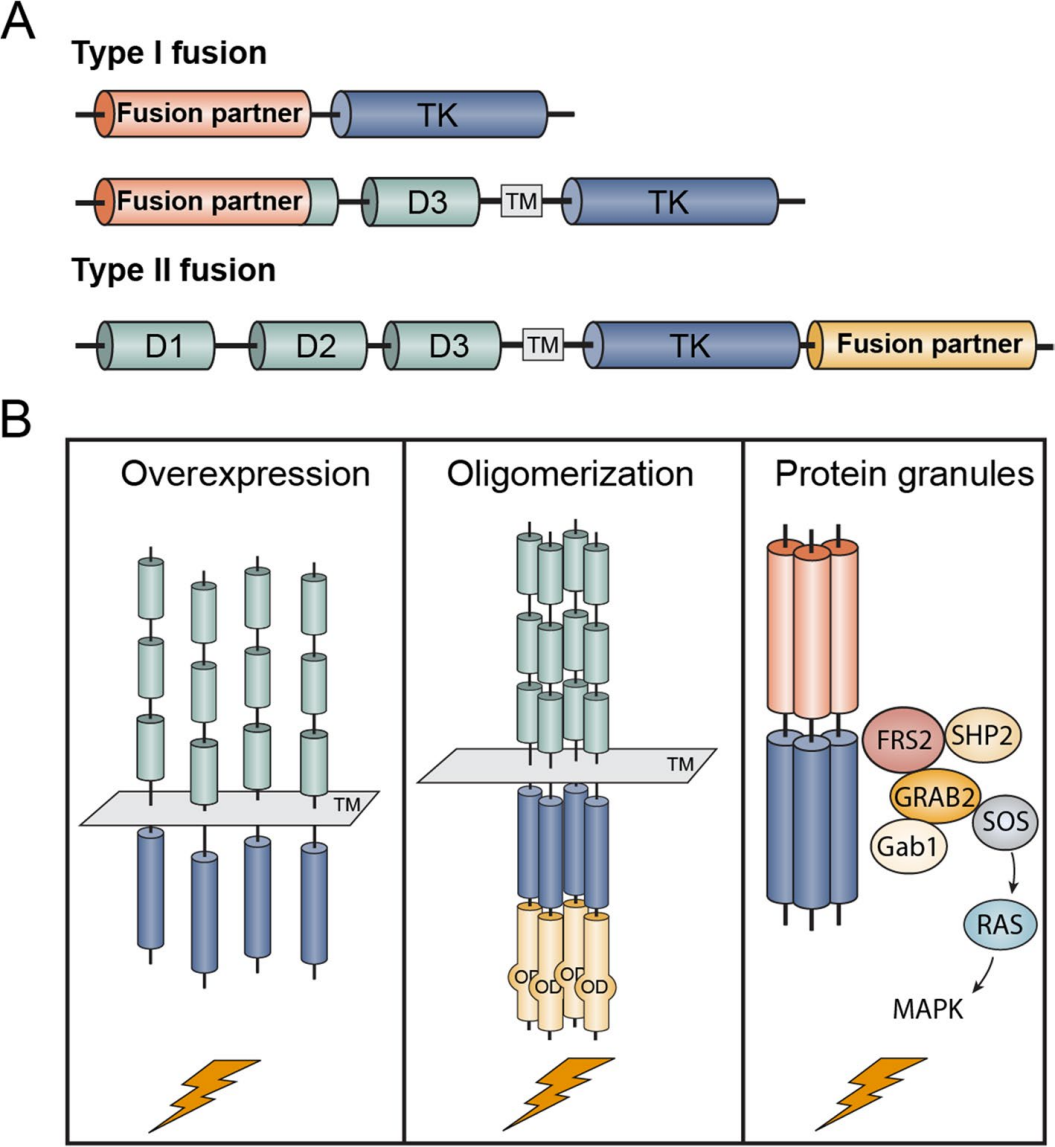
FGFR fusions. **A** Schematic representations of FGFR type I/II fusions. Fusions of FGFR with genes that encode other signaling proteins at N- terminal (type I) or C- terminal (type II) result in release of autoinhibition state and followed by aberrant kinase activation. **B** Potential oncogenic mechanisms of FGFR fusions. Left: fusions produce elevated oncogenic signaling through promoter exchange and FGFR overexpression. Middle: ligand-independent FGFR oligomerization lead to constitutively activation of FGFR kinase mediated by the PPIs through the oligomerization domain (OD) within the fusion partners. Right: FGFR fusion oncoproteins may undergo a higher-order assembly to produce membraneless cytoplasmic protein granules that promote local RAS activation and induce MAPK signaling activation in cancer. TM, transmembrane region; TK, tyrosine kinase domain

Upon ligand-induced receptor dimerization and intracellular kinase A-loop phosphorylation, these self-regulatory switches are subsequently released to turn on the kinase activity. Genetic alterations of FGFRs, including FGFR fusions that eliminate molecular brake or enzyme-substrate repulsion, are proved to be oncogenic. Around 8% of FGFR genetic alterations-related cancers are driven by FGFR gene fusion, which can be classified into type I or type II fusions (Fig. 4A) [18]. In type I fusions, the extracellular and the transmembrane part of the receptors are replaced by the fusion partners. The FGFR kinase domains are forced to dimerization, facilitated by the 5′ fusion partner. In type II fusions, kinase activity is triggered by the fusion at C-terminal regions with the whole receptors remaining intact [44].

A few fusion partners, such as SLC45A3 identified in patients with prostate cancer, can drive overexpression of FGFR2 through promoter exchange (Fig. 4B) [45]. The SLC45A3–FGFR2 fusion contains most of the promoter region of SLC45A3 and only the non-coding region of exon 1, which has the similar oncogenic mechanism to the most famous TMPRSS2–ERG fusion gene existing in more than 50% of prostate cancers [45, 46]. For most cases, the fusion domains provide particular self-association domains that induce ligand-independent dimerization/oligomerization through specific protein-protein interactions (PPIs). These result in constitutive autophosphorylation of the FGFR and aberrant activation of multiple downstream oncogenic signaling cascades in a ligand-independent manner [45]. Those fusion partners include coiled-coil (e.g. FGFR3-TACC3, FGFR2–CCDC6 and FGFR2–CIT), LIS1-homologous (LisH, e.g. FGFR2–OFD1), sterile alpha motif (SAM, e.g. FGFR2–BICC1), IRSp53/MIM homology domain (IMD, FGFR3–BAIAP2L1), and caspase domains (e.g. FGFR2–CASP7) (Fig. 4B) [4, 45].

Besides, Tulpule et al. [47] demonstrated that RTK fusion oncoproteins can form membraneless intracellular protein granules. An array of RTK adaptor and effector molecules and RAS activating proteins are identified at the biomolecular condensates, including GAB1, GRAB2, SHP2, and SOS1. This higher-order protein assembly is crucial for the activation of oncogenic RAS-MAPK signaling (Fig. 4B). Interestingly, the cytoplasmic granule formation may be a general mechanism for oncogenic RTK-mediated signaling activation by FGFR [47]. Thus, drugs to disrupt the nucleation of FGFR membraneless cytoplasmic protein granules may provide opportunities for the treatment of FGFR oncoprotein-driven cancers.

## FGFR gene fusions in cancers

The use of next-generation sequencing approaches for clinical diagnostics greatly promoted the discovery of FGFR molecular alterations in cancers [48, 49]. Fusion events were reported between FGFRs and numerous partners correlated to various cancer progressions (Table 1). FGFR1 fusions correlate to aggressive haematological malignancies and solid tumors, including breast cancer and lung cancer. FGFR2 fusions mainly correlate to cholangiocarcinoma [13, 50]. FGFR3 functions corrupted by translocations are frequently observed in urothelial carcinoma and glioblastoma. FGFR4 fusions are rare, and only some cases were reported in non-small cell lung cancer (NSCLC) [48]. In addition, FGFRs-containing gene fusions are emerging targets for FGFR-targeted cancer therapies.

**Table 1:** FGFR fusion partners

Gene	5′-Gene	3′-Gene	Tumor type	Case reported
FGFR1	ZNF198	FGFR1	Hematopoietic neoplasm	Not reported
	BCR	FGFR1	Hematopoietic neoplasm	Not reported
	FOP1	FGFR1	Hematopoietic neoplasm	Not reported
	CNTRL	FGFR1	Hematopoietic neoplasm	Not reported
	BAG4	FGFR1	Non-small cell lung cancer	1/220
	RHOT1	FGFR1	Breast cancer	1/1019
	NSD3	FGFR1	Breast cancer	2/1019
	FGFR1	HOOK3	Gastrointestinal stromal tumor	1/186
	FGFR1	TACC1	Glioblastoma	1/97
	FGFR1	ZNF703	Breast cancer	1/24
	FGFR1	NTM	Bladder urothelial carcinoma	1/295
	FGFR1	ADAM18	Breast cancer	1/1019
	FGFR1	SLC20A2	Lung adenocarcinoma	1/487
FGFR2	FGFR2	PPHLN1	Intrahepatic cholangiocarcinoma	20/122
	FGFR2	BICC1	Intrahepatic cholangiocarcinoma	2/66
	FGFR2	AHCYL	Intrahepatic cholangiocarcinoma	7/66
	FGFR2	CCAR2	Lung squamous cell carcinoma	1/220
	FGFR2	USP10	Ovarian cancer	1/400
	FGFR2	OFD1	Thyroid carcinoma	1/494
FGFR3	FGFR3	TACC3	Glioblastoma	6/158
	FGFR3	TACC3	Low-grade glioma	1/266
	FGFR3	TACC3	Non-small cell lung cancer	5/220
	FGFR3	TACC3	Bladder cancer	3/121
	FGFR3	TACC3	Head and neck squamous cancer	2/300
	FGFR3	TACC3	Lung squamous cell carcinoma	4/2375
	FGFR3	BAIAP2L1	Bladder cancer	2/46
	FGFR3	TPRG1	Head and neck squamous cancer	1/300
	FGFR3	ELAVL3	Low-grade glioma	1/266
	FGFR3	AES	Prostate adenocarcinoma	1/178

### FGFR1 fusions in haematological malignancies

Myeloid and lymphoid neoplasms with aberrant FGFR1 activities have been classified into a distinct disease group in haematological neoplasms by the World Health Organization in 2008 [51]. This kind of rare but aggressive haematological malignancies are associated with chromosomal translocation of FGFR1 on chromosome 8p11–12, and later are described as 8p11 myeloproliferative syndrome, also known as stem cell leukemia/lymphoma (SCLL). The symptoms include eosinophilia, lymphadenopathy, and lymphoma with subsequent progress to B-cell lymphoma and acute myeloid leukemia [51].

To date, more than 14 FGFR1 fusion partners in hematopoietic neoplasm have been described, and the zinc-finger domain ZNF198 (also known as ZMYM2) on chromosome 13q12 is the most typical partner gene [52–54]. Other neoplasms with chromosomal abnormalities include t(8;22)(p11;q11), t(6;8)(q27;p11) and t(8;9)(p11;q33). These chromosomal abnormalities result in FGFR1 fusion with BCR (breakpoint cluster region) [55], FOP1(FGFR1 oncogenic partner 1) [56], and CNTRL (centrosomal Protein 1) [57], respectively. All these fusion proteins related to haematological malignancies are type I FGFR fusions. These fusion proteins do not have extracellular FGF binding domains, so the dimerization/oligomerization and transphosphorylation of FGFR kinase occur in a ligand-independent manner.

BCR–FGFR1 occurs in stem cell leukemia/lymphoma, which can progress to atypical chronic myeloid leukemia, acute myeloid leukemia, or B-cell lymphoma. In BCR–FGFR1 fusion, BCR functions as a coiled-coil oligomerization domain and promotes oncogenic transformation. Recently, Peiris et al. [55] demonstrated the formation of three interhelical salt bridges by BCR domain contributing to the cellular transforming ability of BCR–FGFR1 fusion. Furthermore, BCR–FGFR1 is a heat shock protein 90 (Hsp90) addicted fusion to evade ubiquitination and proteasomal degradation. Thus, in addition to the kinase domain inhibitors, targeting BCR oligomerization and chaperonin Hsp90 complex can be alternative therapeutic strategies to combat BCR–FGFR1 fusion-positive SCLL.

### FGFR2 fusions in cholangiocarcinoma

Cholangiocarcinoma is a fatal biliary tract cancer. The five-year survival rate of cholangiocarcinoma patients was less than 10%, owing to the limited therapeutic options [58]. Genomic analysis has shown that FGFR2 fusions were identified in 13% ~ 50% of intrahepatic cholangiocarcinoma (iCCA) patients. FGFR2 fusion harboring iCCA shows unique pathologic characteristics, including growing with a tubular anastomosing or intraductal pattern, and lack of stem-like cell markers (CD56 and KIT) [50, 59]. Notably, FGFR2-fusions were rarely observed in perihilar cholangiocarcinoma (PHC) or distal cholangiocarcinoma (DC) [60].

Currently, more than a hundred different FGFR2 fusion protein chimeras have been reported in iCCA. FGFR2–PPHLN1 (Periphilin) is a particularly common type II FGFR fusion chimera in iCCA, resulting from the t(10;12)(q26,q12) translocation. It was identified in nearly 16% of iCCA patients. The C-terminal coiled-coil region derived from PPHLN1 was found to mediate dimerization/oligomerization and favor the oncogenic capability [50, 61]. Indeed, expression of FGFR2-PPHLN1 in HEK293T cells showed robust FGFR phosphorylation and activation of downstream MAPK signaling. NIH3T3 cells transfected with FGFR2–PPHLN1 displayed increased transforming activity in a soft agar assay. In addition, the HUCCT1 cell line overexpressing FGFR2–PPHLN1 obtained increased viability and migratory capacity [50]. Other coiled-coil or sterile alpha motif (SAM) domains containing FGFR2 fusion partners, including coiled-coil domain containing 6 (CCDC6) [62], BicC family RNA-binding protein 1 (BICC1) [63] and adenosylhomocysteinase like 1 (AHCYL) [50] are also found in iCCA patients with frequencies of 3, 6 and 11%, respectively [59]. FGFR2–CCDC6 fusion significantly enhanced tumor cell proliferation and tumorigenesis in an iCCA patient-derived xenograft (PDX) mouse model [62].

### FGFR3 fusions in urothelial carcinoma and glioblastoma

Compared to the high frequency of FGFR2 fusions in cholangiocarcinoma, FGFR3 fusions are more commonly detected in urothelial carcinoma and glioblastoma multiforme (GBM), with fewer cases detected in lung cancer [4, 13]. For instance, FGFR3 genetic alterations are detected in 20–50% of bladder cancer patients, particularly with a high frequency of oncogenic gene fusion FGFR3–TACC3 (the transforming acidic coiled-coil containing protein gene-3) [44, 64]. FGFR3–TACC3 was first described in human glioblastoma (3% cases) and was subsequently found in many other cancers like urothelial carcinoma [65]. The unique feature of oncogenic TACC proteins is a prominent coiled-coil domain at the C-terminus, facilitating kinase transphosphorylation and localization of FGFR–TACC3 to the mitotic spindle leading to chromosomal segregation defects in cancer cells [65]. Of interest, this type II FGFR–TACC3 chimera can activate MAPK and JAK-STAT signaling pathways but not PLCγ-dependent signaling because of the lack of PLCγ docking site at Tyr760 [66].

Although two direct intracellular substrates of FGFRs (FRS2 and PLCγ) have been known for decades, the downstream effectors of FGFR fusions have not been clearly elucidated. Recently, Frattini and colleagues [17] identified PIN4, as a novel substrate of the FGFR3–TACC3 fusions, was required for reactive oxygen species (ROS)-mediated induction of peroxisome proliferator-activated receptor gamma coactivator 1-alpha (PGC1α) and tumor growth. Compared to its kinase-dead form (K508M mutant), FGFR3–TACC3 increased PIN4 phosphorylation at Tyr122, thereby promoting mitochondrial respiration and ATP production and tumor progression [17]. This finding highlights the downstream substrate as a therapeutic opportunity for the treatment of tumors with FGFR fusions. However, due to the diversity of FGFR gene fusion types, it should be further studied whether those effectors such as PIN4 work as a common node.

## FGFR-targeted inhibitors for FGFR fusion-harboring cancer therapy

Chromosomal translocations that generate in-frame oncogenic gene fusions are remarkable examples of the success of targeted cancer therapies. Although FGFR fusions are relatively rare, they have become novel druggable targets. According to the records on the ClinicalTrials.gov website, there are currently 27 FGFR-targeted inhibitors in clinical trials for cancer therapy. Those trials have included FGFR fusion-addicted cancers in the subsets of patients with cholangiocarcinoma, urothelial carcinoma, glioma, breast cancer, lung cancer or lymphoma (Table 2).

**Table 2:** Overview of clinical trials involving FGFR fusions

Inhibitor	IC_50_	Clinical trial ID	Genetic alterations	Cancer type	Phase	Status
**First-generation inhibitors**						
Derazantinib (ARQ-087)	FGFR1 (4.5 nM) FGFR2 (1.8 nM) FGFR3 (4.5 nM) FGFR4 (34 nM)	NCT01752920	FGFR genetic alterations including FGFR2 gene fusion	Advanced solid tumors	I/II	Completed
		NCT03230318	FGFR2 gene fusion, mutation or amplification	Cholangiocarcinoma	II	Recruiting
		NCT04045613	FGFR genetic aberrations	Urothelial cancer	I/II	Recruiting
Ponatinib	FGFR1 (2.2 nM) FGFR2 (1.6 nM) FGFR3 (18.2 nM) FGFR4 (7.7 nM)	NCT02265341	FGFR2 fusions	Biliary cancer	II	Completed
		NCT02272998	FGFR alterations including fusions	Advanced solid tumor	II	Recruiting
**Second-generation inhibitors**						
Pemigatinib (INCB054828)	FGFR1 (0.4 nM) FGFR2 (0.5 nM) FGFR3 (1.2 nM) FGFR4 (30 nM)	NCT03656536	FGFR2 rearrangement.	Cholangiocarcinoma	III	Recruiting
		NCT04003610	FGFR3 mutation or rearrangement	Urothelial carcinoma	II	Active
		NCT04258527	FGF/FGFR alterations	Advanced malignancies	I	Active
		NCT04096417	FGFR alterations	Colorectal cancer	II	Recruiting
		NCT02872714	FGF/FGFR alterations	Urothelial carcinoma	II	Active
		NCT03822117	FGFR mutations or fusions	Solid tumor	II	Recruiting
		NCT04003623	FGFR mutations or translocations	Solid tumors	II	Recruiting
		NCT03011372	FGFR1 rearrangement	Myeloid/lymphoid neoplasms	II	Recruiting
Erdafitinib (JNJ-42756493)	FGFR1 (2.0 nM) FGFR2 (2.0 nM) FGFR3 (4.0 nM) FGFR4 (6.3 nM)	NCT02365597	FGFR genomic alterations.	Urothelial cancer	II	Recruiting
		NCT02465060	FGFR amplification mutation or fusion	Solid tumors, lymphomas, or multiple myeloma	II	Recruiting
		NCT03827850	FGFR genetic alterations	Non small cell lung carcinoma	II	Recruiting
		NCT03390504	FGFR gene aberrations	Urothelial cancer	II	Recruiting
		NCT04083976	FGFR mutations and gene fusions.	Advanced solid tumors	II	Recruiting
Infigratinib (BGJ398)	FGFR1 (0.9 nM) FGFR2 (1.4 nM) FGFR3 (1.0 nM) FGFR4 (60 nM)	NCT03773302	FGFR2 gene fusions/translocations	Cholangiocarcinoma	III	Recruiting
		NCT04424966	FGFR3-TACC3 translocation	High-grade glioma	I	Recruiting
		NCT02150967	FGFR genetic alterations	Cholangiocarcinoma	II	Recruiting
		NCT04197986	FGFR3 genetic alterations including fusion	Urothelial carcinoma	III	Recruiting
		NCT04233567	FGFR1–3 gene fusions or other genetic alterations	Solid tumors	II	Recruiting
Debio1347 (CH5183284)	FGFR1 (9.3 nM) FGFR2 (7.6 nM) FGFR3 (22 nM) FGFR4 (290 nM)	NCT03834220	FGFR1–3 fusions	Solid Tumors	II	Active
AZD4547	FGFR1 (0.2 nM) FGFR2 (1.8 nM) FGFR3 (2.5 nM) FGFR4 (165 nM)	NCT02824133	FGFR-TACC gene fusion	Malignant glioma	I/II	Completed
**Third-generation inhibitors**						
Futibatinib (TAS-120)	FGFR1 (3.9 nM), FGFR2 (1.3 nM), FGFR3 (1.6 nM) FGFR4 (8.3 nM)	NCT04093362	FGFR2 gene rearrangements	Advanced cholangiocarcinoma	III	Not yet recruiting
		NCT04189445	FGFR1–4 rearrangements	Solid tumors/ myeloid or lymphoid neoplasms	II	Recruiting
		NCT02052778	FGFR fusion or activating mutation or amplification	Advanced solid tumors	I/II	Active,

### First-generation FGFR inhibitors

The first-generation FGFR inhibitors (e.g., derazantinib, ponatinib, lucitanib, dovitinib, lenvatinib and nintedanib) were non-selective inhibitors against multiple tyrosine kinases (e.g., PDGFRs, VEGFRs, KIT, and RET) owing to the high similarity at the ATP binding site of the intracellular kinase domains among RTK family. There are few ongoing clinical trials to use first-generation inhibitors to treat cancer patients with FGFR fusions.

**Fig. 5:**
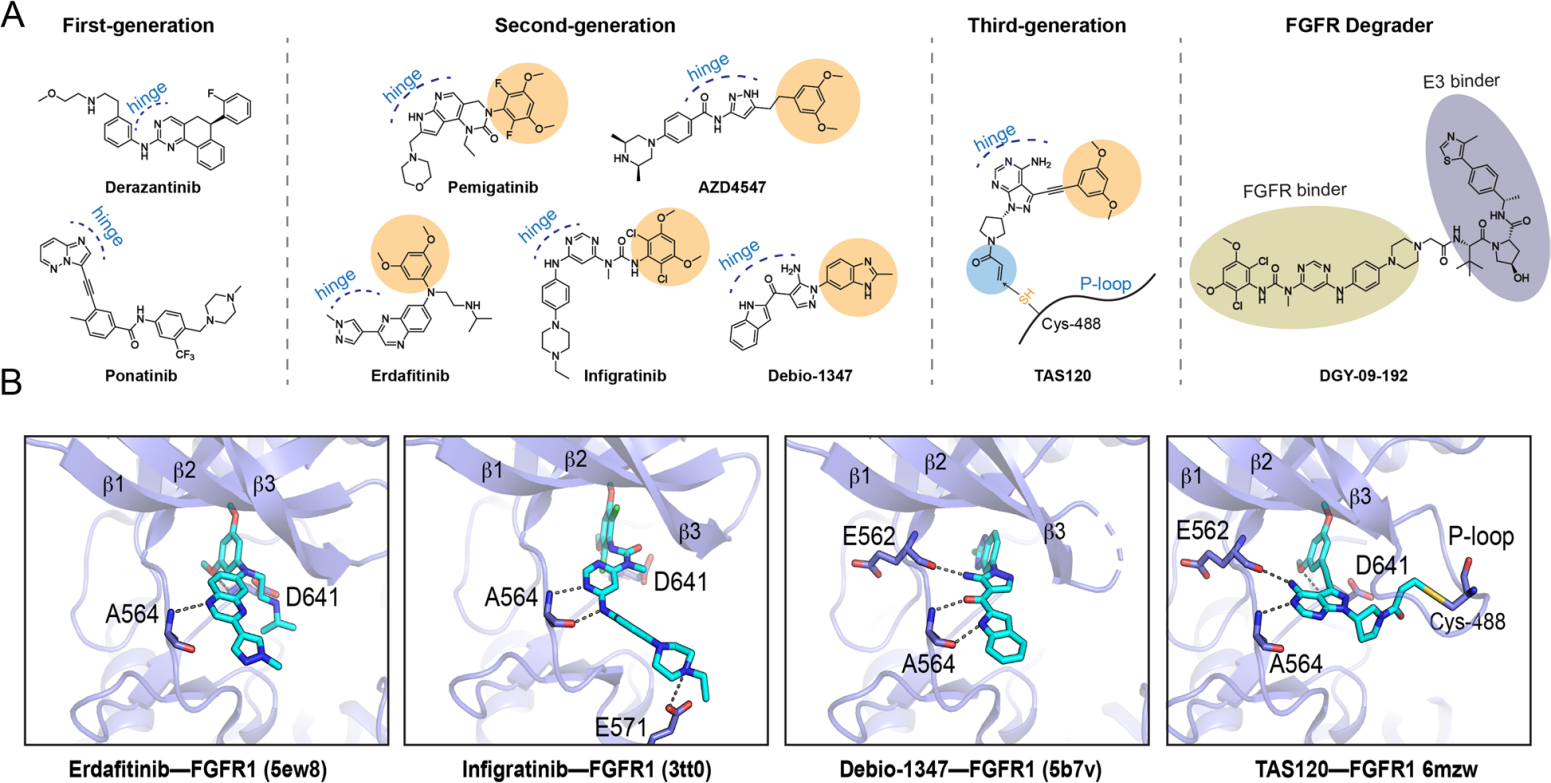
FGFR inhibitors. **A** Chemical structures of selected FGFR inhibitors and PROTAC. The hinge binding region and FGFR hydrophobic pocket binding group are highlighted. **B** Structures of second- and third-generation drug-FGFR complexes, including erdafitinib (5ew8), infigratinib (3tt0), debio-1347 (5b7v), and TAS120 (6mzw) in complex with FGFR1 kinase domain

Derazantinib (ARQ 087) is a 5,6 dihydrobenzo[*h*]quinazolin-2-amine derivative inhibitor against FGFR2 (1.8 nM), FGFR1 (4.5 nM), and FGFR3 (4.5 nM) kinases (Fig. 5). The crystal structure of the FGFR2-ARQ069 complex shows that the aminopyrimidine scaffold contributes to hinge interaction, and the hydrophobic part of the compound stabilizes the G-loop conformation through non-polar interactions [67]. In FGFR2 transfected Ba/F3 cell lines, derazantinib displayed potent inhibition of FGFR2 fusions, including FGFR2-CCDC6, FGFR2-BICC1, TEL-FGFR2, FGFR3-BAIAP2L1 and FGFR2-AAF3 with GI_50_ values between 39.9 nM and 1121 nM. Derazantinib also showed strong tumor inhibition in FGFR2 fusion-driven tumor xenograft models [68]. In the phase I/II study (NCT01752920), derazantinib exhibited promising antitumor activity in subjects with progressed iCCA harboring FGFR2 gene fusions after systemic chemotherapy. The overall response rate (ORR), disease control rate (DCR) and estimated median progression-free survival (PFS) were 20.7, 82.8% and 5.7 months, respectively. A following larger pivotal trial of derazantinib in iCCA is under recruitment (NCT03230318) [69, 70].

### Second-generation FGFR inhibitors

Multi-kinase FGFR inhibitors can result in a series of adverse effects due to low specificity and potency, such as cardiovascular and liver toxicities, proteinuria, and hypertension [71, 72]. Thus, second-generation FGFR selective inhibitors (e.g., erdafitinib, pemigatinib, infigratinib, Debio1347, rogaratinib, and AZD4547) are emerging to lower the risk of adverse effects and improve clinical outcomes (Fig. 5). Two selective FGFR-TKIs (erdafitinib and pemigatinib) have been approved by the U.S. FDA for the treatment of FGFR-driven cancers.

Erdafitinib is a pan-FGFR inhibitor with a quinoxaline core which was granted accelerated approval by the FDA for the first-line treatment of urinary bladder tumors with FGFR2/3 mutant or FGFR2/3 fusion and the second-line treatment of metastatic or unresectable urothelial carcinoma. In the phase II study (NCT02365597) that enrolled 99 patients with advanced urinary bladder cancers harboring FGFR3 point mutation or FGFR2/3-containing fusions receiving erdafitinib, the ORR was 40% with 3% of the patients getting a complete response, and the patients had a median PFS duration of 5.5 months and median overall survival (OS) duration of 13.8 months. In the subgroup of 25 patients with FGFR fusions, the ORR was 16%. Particularly, 36% (4/11) of FGFR3–TACC3 fusion carrying patients had a response to erdafitinib treatment [73, 74].

Pemigatinib is a tetra-azatricyclotridecatetraene derivative with a highly selective inhibition against FGFR1–3. FGFR2 fusions have been considered a promising therapeutic target for cholangiocarcinoma in clinical practice after the FDA-accelerated approval of pemigatinib to treat cholangiocarcinoma patients carrying FGFR2 fusions or rearrangements. The efficacy of pemigatinib was evaluated in a phase II study (NCT02924376) among 107 cholangiocarcinoma patients harboring chimeric FGFR2 proteins. The ORR was 35.5% (38/107) with a 2.8% (3/107) complete response rate. Notably, no complete or partial responses were observed in patients with other types of FGFR alterations or without FGFR alterations [75, 76]. These encouraging data demonstrate the potential benefit of pemigatinib in cholangiocarcinoma patients with FGFR2 fusions or rearrangements. A phase III FIGHT-302 study (NCT03656536) is ongoing to compare the efficacy of pemigatinib versus chemotherapy as first-line treatment for unresectable or metastatic cholangiocarcinoma with FGFR2 alterations.

Infigratinib (BGJ398) is a dianimopyrimidine derivative that selectively targets FGFR1–3. In a phase II study (NCT02150967) of infigratinib in advanced refractory or metastatic cholangiocarcinoma with chimeric FGFR2 fusions or other FGFR alterations, all responsive tumors carry FGFR2 fusions. The ORR and the disease control rate (DCR) in patients with FGFR2 fusions were 18.8% (9/48) and 83.3% (40/48), respectively. Reduced target lesion size was observed in 75% (36/48) of FGFR2 fusion-positive patients. Currently, infigratinib is in a phase III study (NCT03773302) as first-line treatment for patients with cholangiocarcinoma harboring FGFR2 gene translocations, and in a phase I study (NCT04424966) in patients with high-grade glioma carrying FGFR3–TACC3 fusions [77]. Another clinical trial (NCT02824133) evaluating the efficacy of AZD4547 (FGFR1–3 inhibitor) in glioma patients with FGFR3–TACC3 fusion is under recruitment [78]. A phase I dose-escalation trial (NCT03834220) using another ATP-competitive FGFR1–3 inhibitor, Debio 1347 (CH5183284), also reported preliminary evidence of antitumor activity in several tumor types, including iCCA [79].

### Third-generation FGFR inhibitors

Although second-generation FGFR inhibitors showed promising antitumor activity in patients with FGFR fusions, acquired resistance occurred due to the emergence of secondary mutations in the FGFR kinase domain [80, 81]. Specifically, clinically observed mutations including N550K, V565F, L618V, and K660M are resistant to infigratinib treatment, whereas N550K, L618V, and K660M mutations confer principal resistance to Debio 1347 [82].

Irreversible kinase inhibitors (e.g., osimertinib, ibrutinib, and neratinib) have been proven feasible in multiple cancers and approved by the FDA for the treatment of EGFR-driven NSCLC, lymphomas, and HER2-positive breast cancer [83]. Futibatinib, a pyrazolo[3,4-*d*]pyrimidine derivative, is designed to covalently bind to a highly conserved cysteine residue (Cys488 in FGFR1c) within the P-loop of FGFR kinase, thus prolonging the pharmacodynamic duration (Fig. 5). Preliminary results from a phase I trial (NCT02052778) of futibatinib in advanced refractory tumors determined an ORR of 25% (7/28) and a DCR of 78.6% in patients with ICC carrying chimeric FGFR2 proteins, including selected patients who had experienced prior therapy with second-generation FGFR inhibitors.

Recently, Goyal et al. [82] reported the results of futibatinib in iCCA patients with FGFR2 translocation and disease progression upon infigratinib or Debio 1347 treatment. Futibatinib effectively overcame multiple secondary FGFR2 resistance mutations and showed clinical benefits in infigratinib or Debio 1347 resistant iCCA patients. These data support that strategically sequencing therapies with anti-FGFR molecules could benefit iCCA patients with FGFR2 fusion. Currently, futibatinib is in a multinational, randomized phase III clinical study (NCT04093362) to assess the efficiency and safety of futibatinib as first-line therapy for advanced or recurrent unresectable iCCA patients with FGFR2 gene rearrangements.

### FGFRs degraders

Despite major progress in the discovery of selective and potent FGFR inhibitors over the past decade, the long-term value of these drugs in cancer treatment has been hindered by the quick onset of acquired resistance. While the third-generation covalent inhibitor futibatinib is effective against certain FGFR mutants, it fails to overcome the gatekeeper mutation [82]. Tumor cells may acquire resistance to irreversible inhibitors like futibatinib by mutating the cysteine residue, a common resistance mechanism previously described for EGFR [84] and BTK [85] covalent inhibitors. Small molecule-induced protein degradation is an emerging strategy in the field of drug discovery. Event-driven proteolysis targeting chimeras (PROTACs) can avoid mutation-related resistance based on the unique degradation mechanism [86]. To test whether FGFRs and their fusion variants are degradable targets, Du et al. [87] developed a low nanomolar PROTAC for FGFRs degradation, DGY-09-192 (Fig. 5). This heterobifunctional molecule showed dose-dependent degradation of FGFR fusion proteins in both CCLP-1-FP and ICC13–7 cells, expressing the FGFR2-PHGDH and FGFR2-OPTN fusion, respectively. In the CCLP1-FGFR2-PHGDH xenograft model, DGY-09-192 at 20 or 40 mg/kg can reduce both FGFR2-PHGDH protein levels and phosphorylation of downstream molecules in vivo. However, DGY-09-192 still has some limitations, i.e., it degrades all FGFR isoforms. Further optimization will be necessary to improve selectivity for a particular FGFR or fusion before reaching the clinic. Besides, we envision that the discovery of degraders towards the non-FGFR part of the oncogenic fusions, such as TACC3 and PHGDH, should be also interesting and promising.

## Conclusion and perspective

Based on the results from clinical trials, tumors with genetic alterations of FGFRs would respond to FGFR inhibitor therapies. In this regard, targeted therapies for FGFR fusion-driven tumors offer an efficient therapeutic strategy in these cancer types. The benefits of FGFR targeting therapy in subsets of fusion-positive patients with haematological malignancies, iCCA, lung cancer, urothelial carcinoma and glioblastoma have been widely proved in clinical trials. Currently, most FGFR fusion-related clinical studies are focused on FGFR2, while there are relatively few clinical studies on FGFR1 and FGFR3 gene fusion. In this April, FDA has granted a breakthrough therapy designation to futibatinib to treat iCCA patients that harbor FGFR2 gene rearrangements or fusions. However, some challenges still exist, such as patient selection, molecule basis study of FGFR fusions, acquired resistance of FGFR inhibitors, and management of adverse events.

FGFR fusions are relatively rare genetic alterations. To improve the clinical benefits of FGFR inhibitor treatment, a more refined patient selection strategy is required. Although many methods effectively detect single nucleotide variants and copy numbers, few methods are accurate for FGFR gene fusion detections due to the complex nature of fusions [88]. Developing diagnostic tests such as whole-genome sequencing (WGS) and immunohistochemical (IHC) staining for the detection of FGFR genetic alterations have the potential to perform clinical trials specifically for patients with rare fusions.

Oncogenic partners contain multimerization motifs, which are generally assumed to constitutively increase their kinase activity by promoting kinase transphosphorylation. However, the structural basis whereby the N/C-terminal fusion partners drive kinase oligomerization is poorly understood. With the recent revolution in structural study tools such as cryoEM, unraveling higher-order FGFR fusion protein assemblies and the cytoplasmic protein granules is becoming more feasible [89]. The better understanding of the molecule basis of FGFR fusion-induced molecule assembly will, in turn, aid in the drug discovery for FGFR fusion-positive cancers.

Despite the promising results of FGFR inhibitors in clinical trials, acquired resistance limits the response duration to anti-FGFR agents. In particular, gatekeeper mutations (e.g., V564F in FGFR2) arise as the common mechanism of acquired resistance after pemigatinib and infigratinib treatment. Although irreversible inhibitors such as futibatinib can overcome those resistant mutations, the cysteine mutation might still occur in the clinical trial [84]. Alternatively, developing FGFR kinase allosteric inhibitor or specific fusion partner inhibitors may avoid acquired mutations within the kinase domain. Currently, several TACC3-targeting inhibitors (e.g., KHS101, BO-264) have been considered as novel anticancer drug candidates [90, 91]. In addition, developing FGFR-targeting PROTACs can directly degrade FGFR and fusion partners, which may avoid inhibitor-induced acquired mutation. Anyway, the important role of FGFR fusions should be taken as a key consideration in the drug design and development of FGFR-TKI molecules for clinical studies.

Inhibitors disrupting the physiological functions of FGF/FGFR signaling lead to the unique spectrum of on-target side-effects. Notably, these inhibitors block the FGF23-FGFR1-αKlotho endocrine signaling, leading to phosphate homeostasis disorders such as hyperphosphataemia, which occur in most patients after drug treatment. Strategies such as co-administration with phosphate binders or intermittent dosing have been taken in the clinical trials to control serum phosphate elevation and avoid dysregulation of the endocrine system [92]. Other common FGFR inhibition-related toxic events include asthenia, alopecia, hyponatraemia, skin and eye dryness, and nail toxicities [93]. Thus, more efforts should be spurred to constantly refine and enhance the clinical management of FGFR inhibition-associated adverse effects.

In conclusion, the awareness of the important role of FGFR fusions has significantly boosted the development of FGFR inhibitors. A comprehensive and deep understanding of FGFR fusion proteins would definitely contribute to the FGFR-targeting drug discovery and benefit cancer patients carrying FGFR fusions.

## Data Availability

Data are available upon reasonable request to the corresponding author.

## Abbreviations

A-loop: Activation loop
AHCYL: Adenosylhomocysteinase like 1
AKT: Phosphoinositide 3- kinase (PI3K)–protein kinase B
BICC1: BicC family RNA-binding protein 1
CCDC6: Coiled-coil domain containing 6
CML: Chronic myeloid leukemia
DCR: Disease control rate
FGFRs: Fibroblast growth factor receptors
FRS2α: FGFR substrate 2α
GRB2: Growth factor receptor-bound protein 2
HBS: Heparan binding site
HSPGs: Heparan sulfate proteoglycans
iCCA: Intrahepatic cholangiocarcinoma
JM: Juxtamembrane
MAPK: Mitogen-activated protein kinase
NSCLC: Non-small cell lung cancer
ORR: Overall response rate
PGC1α: Peroxisome proliferator-activated receptor gamma coactivator 1-alpha
PLCγ: Phospholipase Cγ
PKC: Protein kinase C
PROTACs: Proteolysis targeting chimeras
ROS: Reactive oxygen species
RTKs: Receptor tyrosine kinases
SAM: Sterile alpha motif
SCLL: Stem cell leukemia/lymphoma
SHP2: SH2-containing tyrosine phosphatase
